# Supplementary material for: Characterizing cell-type spatial relationships across length scales in spatially resolved omics data
Source: Nat Commun. 2025 Jan 3;16:350. doi: 10.1038/s41467-024-55700-1 (PMC11699133; doi:10.1038/s41467-024-55700-1)
Supplement: Supplementary file 3 — Supplementary Software 1 [file 41467_2024_55700_MOESM3_ESM.zip › CRAWDAD/plots/seqfish/S5_seqfish_haem_ripleys_facet.pdf]

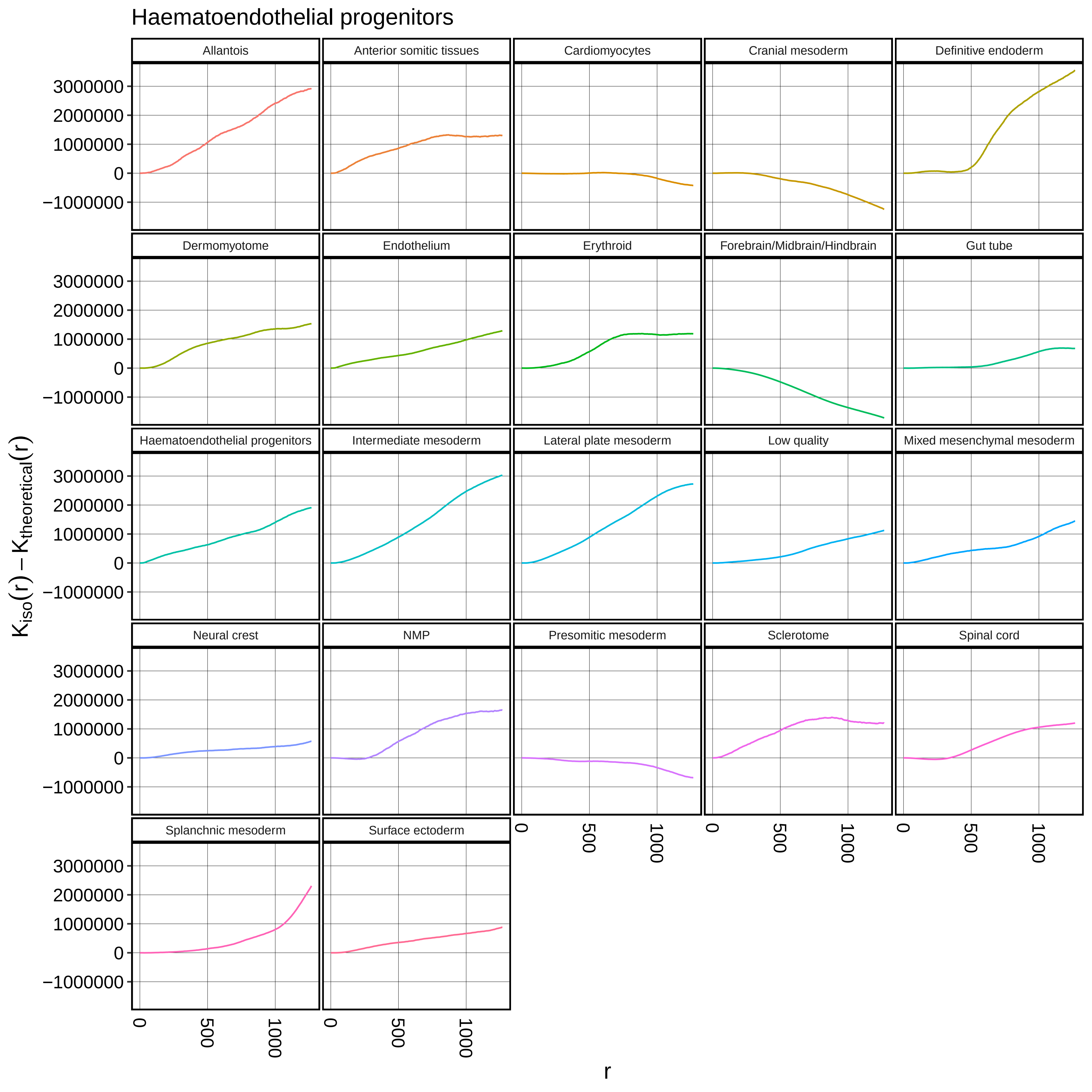

Haematoendothelial progenitors
Allantois
Anterior somitic tissues
Cardiomyocytes
Cranial mesoderm
Definitive endoderm
Dermomyotome
Endothelium
Erythroid
Forebrain/Midbrain/Hindbrain
Gut tube
Haematoendothelial progenitors
Intermediate mesoderm
Lateral plate mesoderm
Low quality
Mixed mesenchymal mesoderm
Neural crest
NMP
Presomitic mesoderm
Sclerotome
Spinal cord
Splanchnic mesoderm
Surface ectoderm
3000000
2000000
1000000
0
−1000000
0
500
1000
$K_{iso}(r) - K_{theoretical}(r)$
r
